# Mock Wards: Incorporating a Theoretical Framework to Create a Blended Virtual and In-Person Clinical Reasoning Education Platform

**DOI:** 10.7759/cureus.64954

**Published:** 2024-07-19

**Authors:** Myles Benayon, Lekhini Latchupatula, Muqtasid Mansoor, Etri Kocaqi, Arden Azim, Matthew Sibbald

**Affiliations:** 1 Internal Medicine, McMaster University, Hamilton, CAN; 2 Medicine, McMaster University, Hamilton, CAN; 3 Cardiology, McMaster University, Hamilton, CAN

**Keywords:** in-person learning, virtual learning, internal medicine, clinical reasoning, medical education

## Abstract

Introduction

The coronavirus 2019 pandemic highlighted virtual learning (VL) as a promising tool for medical education, yet its effectiveness in teaching clinical reasoning (CR) remains underexplored. Past studies have suggested VL can effectively prepare students for clinical settings. Informed by the Milestones of Observable Behaviours for CR (MOBCR) and whole-case theoretical frameworks, the Mock Wards (MW) program was created using a novel blended in-person learning (IPL) and VL platform. MW consisted of case-based small-group formats for medical students interested in learning approaches and differentials to commonly encountered presenting symptoms and diagnoses in internal medicine. This study sought to use MW’s blended design to qualitatively analyze CR development and compare its utility between VL and IPL.

Methods

Qualitative analysis was conducted using in-depth semi-structured interviews with first-year pre-clerkship medical students (n = 8) who completed the MW program and participated in the study. The interview guide was informed by the MOBCR framework. Interview transcripts were analyzed using a directed qualitative content analysis approach. Translational coding and HyperRESEARCH^TM^ (Researchware, Inc., Randolph, MA) software-generated mind maps guided the theme development.

Results

Three overarching themes were constructed: (1) tailoring pedagogical frameworks to learning modalities, (2) learning through interactivity, and (3) balancing accessibility with learner engagement. Participants emphasized that teaching CR skills is modality-specific and not fully interchangeable, with IPL being superior in facilitating social cohesion, non-verbal communication, and feedback. In contrast, VL required structured approaches and relied more on verbal communication and pre-made digital materials. IPL also enhanced interactivity, peer relationships, and spontaneous communication, whereas VL faced challenges such as social awkwardness and technological constraints hindering effective collaboration. VL provided superior accessibility to facilitate distributed learning and management of concurrent academic obligations.

Conclusion

The MW-blended platform highlights the importance of focusing on modality-tailored pedagogies, emphasizing group interactability, and balancing VL accessibility against decreased engagement within the IPL environment when teaching CR skills. Blended education models may benefit from a scaffolding approach, using IPL as a prerequisite to VL to improve CR development and alignment within a learner’s zone of proximal development.

## Introduction

Clinical reasoning (CR) is a multifaceted capability where clinicians systematically observe, gather, and analyze data to diagnose and determine treatment plans. CR involves conscious and unconscious thought processes that interact with various contextual elements, such as a patient's specific medical history, goals of care, and healthcare environment. Undergraduate medical programs cover fundamental aspects of diagnosis, such as taking patient histories, conducting physical exams, and forming differential diagnoses. However, the deeper skills and behaviors crucial for proficient CR are often learned informally through hands-on practice and guidance from experienced clinicians [1]. In many medical schools, CR is limited to being an implicit component of the curriculum that is not explicitly taught [2]. Research has shown that CR strategies differ significantly between novice and expert learners, as experts often rely on heuristics and pattern recognition, whereas novices require more structured, analytical approaches. If medical education is to shift towards a more explicit approach to teaching CR, it becomes crucial to consider how these strategies evolve with experience and to tailor educational interventions accordingly [3].

CR has an important role in medical education, particularly in pre-clinical years, yet evidence has suggested inadequate CR development through existing curricula [4]. Students often cite the pre-clinical stage as the most challenging to build CR skills due to its separation from the clinical setting and limited exposure to diverse opportunities, resources, and medical specialties [4,5]. Furthermore, surveys have revealed that students often enter clinical rotations lacking a robust grasp of essential CR skills [1]. Virtual learning (VL) has been shown to help maximize exposure to patient cases and aid in knowledge consolidation to improve CR skills [4].

Due to the coronavirus 2019 pandemic, medical students experienced significant disruptions to medical education during pre-clerkship and clerkship when in-person learning (IPL) was limited. The increased demand for VL accelerated the integration of such modalities within existing medical curricula. A 2020 pilot educational platform at McMaster University called Virtual Wards (VW) was initiated during the pandemic to increase medical learning opportunities through a VL format. VW provided optional small-group tutorial sessions led by resident teachers that taught high-yield internal medicine approaches in a case-based format to 166 medical students [6]. This instructional approach emphasizes near-peer teaching, a common practice in medical education where individuals slightly more advanced in their training provide guidance and education to those with less experience [7].

Post-hoc analysis of the VW initiative revealed that 33 (72%) of the 46 surveyed medical students found the pilot program extremely helpful, and 43 (93%) students agreed VW would be a beneficial complementary learning tool, even without curriculum disruptions from the pandemic. This model was low-cost and highly accessible, allowing for potential utilization in various other educational contexts. While the study did not explicitly assess CR, the authors suggested their modality may help foster CR skills in a post-pandemic era [6].

The development of novel virtual modalities to teach CR should be informed by existing CR theoretical frameworks to create a specific teaching method and evaluate whether the intended modalities are efficacious in developing CR skills. The whole-case approach to teaching CR is a method in which all information about a patient case is presented at once and available to the student throughout the problem-solving process [8]. This contrasts with the serial-cue approach, where information is given sequentially. The whole-case approach is thought to be more effective in teaching early-stage medical students by reducing the cognitive load on working memory [8].

Acquiring CR abilities is challenging because they encompass a set of skills rather than a single, well-defined ability. These skills require synthesizing the complexities of medical issues and applying existing knowledge and capabilities to new situations. The Milestones of Observable Behaviors CR (MOBCR) framework offers a methodology to assess the specific components of CR in the early stages of developing these skills among medical students. The MOBCR framework describes CR as a constellation of the following individual skills: identifying pertinent facts, collecting and recording data for differential diagnoses, developing hypotheses, rationalizing hypotheses, providing feedback, participating in group problem-solving, asking questions to address knowledge gaps, citing relevant research sources, reflecting on cognitive errors, seeking insight into personal weaknesses, recognizing diverse group perspectives, and documenting personal and peers' work [5].

VL provides advantages when IPL is unavailable but may not be superior to IPL problem-based tutorials [2]. VL has also been thought to decrease social interaction and lead to disruptions in learning through technical difficulties [9]. The benefits of increased interactivity include more audience engagement and deeper learning. Past evidence has suggested VL opportunities are effective supplementary teaching modalities to existing medical curricula [2]. The effects of VL and the development of CR skills in medical school require further investigation.

We synthesized the MOBCR, near-peer teaching, and whole-case approaches to launch a novel educational platform for medical students called Mock Wards (MW) [5,7-8]. This blended, optional CR learning opportunity consisted of both in-person and virtual small-group, case-based tutorials led by internal medicine residents as the teachers. MW sought to supplement existing medical curricula at McMaster University. This study aimed to use MW’s blended framework to qualitatively analyze CR development and compare its utility between VL and IPL among pre-clinical medical students, a stage in medical training when CR development is most challenging [4,5].

This article was previously presented as an abstract at the 2024 McMaster University Internal Medicine Resident Research Day on May 15, 2024.

## Materials and methods

A qualitative study was conducted using in-depth, semi-structured interviews to explore pre-clerkship medical students’ perceptions of CR skills development through MW.

Context, sampling, and informants

The MW-blended education platform consisted of six sessions at McMaster University, consisting of three virtual and three in-person sessions. Each of the six sessions had a different topic. The authors of this study strongly recommended that resident teachers focus on case-based approaches, such as clinical approaches to chest pain, shortness of breath, hypotension, anemia, diarrhea, and acute kidney injury. Each resident teacher was responsible for creating, selecting, and sharing their chosen teaching materials and resources for MW. Resident teachers were recommended to keep these sessions interactive instead of solely didactic and promote their students to use CR to answer the presented clinical problems or questions.

MW sessions occurred between April and June 2023. During these months of training, first-year medical students covered the majority of foundational pre-clerkship learning objectives in their undergraduate medical education but had not yet begun their clinical rotations, which started in July 2023. This time period was considered optimal for learners to be within a zone of proximal development, where they can integrate all their pre-clerkship didactic content into approaches and enhance their CR skills [10,11].

First-year medical students who were willing to participate in this study must have attended at least one virtual and one in-person MW session and had at least one month of clinical work after completing MW to fulfill inclusion criteria. Those who were in remediation or no longer enrolled in McMaster’s medical school were excluded from the study.

A maximum variation sampling purposive recruitment strategy was used [12]. An email was sent to all MW students to recruit participants for the study. Students who were willing to participate were requested to confirm the inclusion and exclusion criteria above. Out of the 131 medical students who participated in MW, eight volunteered to participate in the study.

Research team

Five members of the team (MS, LL, MB, MM, EK) were involved in reviewing and analyzing the interview transcripts. Each member brings different perspectives and professional experiences relating to medical education and teaching. MS is the Associate Dean at McMaster Undergraduate Medicine and has a Ph.D. in Education from Maastricht University. MS is also an interventional cardiologist at Hamilton Health Sciences, cardiology residency program director, and researcher. LL and MB are current postgraduate internal medicine residents with MDs from McMaster University and a strong interest in medical education with previous publications on the topic. They are also clinical skills preceptors for undergraduate medicine students and participated in VW as medical students. LL and MB also took part in the executive team that brought the McMaster University Book2Bedside conference, a medical education conference for medical students, to the virtual environment during the coronavirus 2019 pandemic. MM is a second-year medical student at McMaster University with also a strong interest in medical education. EK is a current postgraduate internal medicine resident with an MD from McMaster University. She has qualitative research and simulation experience. The team understood that each member would hold various interpretations that are grounded in individual expertise, research work, and life experience, but everyone held the same understanding of clinical reasoning as informed by the MOBCR framework [5]. The team approached the collected data neutrally without support for any particular interpretation of the results.

Interview guide

The study’s interview guide was informed by the MOBCR rubric and framework [5]. A semi-structured interview format was conducted, and the interview was informed by open-ended question models [12,13]. The inclusion criteria were verified again at the start of the interview. Afterward, the participants were asked how VL sessions reflected the process of obtaining CR skills during IPL sessions. Interviews then focused on how effectively VL facilitated group problem-solving. Subsequently, the questions centered on the challenges and barriers to learning within the virtual context in relation to either CR skill acquisition or interpersonal interactions. Lastly, interviewers asked several questions comparing how the process of developing one or more of the domains of CR was impacted between the VL and IPL contexts. An in-depth interview guide with the specific questions asked under each section can be found in Appendix A.

Data collection

Eight interviews were completed over Zoom® by the same research team members between November and December 2023 to ensure consistency across interviews. Participants' written consent was obtained before their interview dates. Participants were provided with a small 20 Canadian dollar gift certificate to compensate for their time.

Interviews were audio-recorded, transcribed verbatim, and subsequently reviewed by MB and LL to ensure anonymity and accuracy. Afterward, interview transcripts were uploaded into HyperRESEARCH™ (Researchware, Inc., Randolph, MA), a qualitative analysis research software [14].

Data analysis

**Figure 1:**
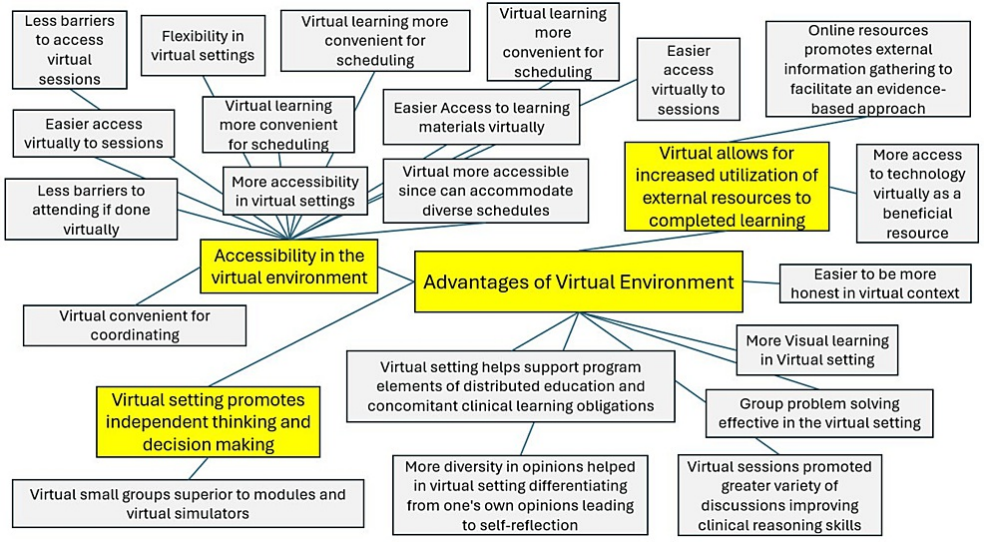
A mind map organizing the findings related to the advantages of the virtual learning environment, which were based on interview transcripts from the eight study participants. Large categories are enclosed in yellow boxes, with branches illustrating the connections between these central categories and their related findings, which are displayed in grey boxes. This mind map was originally created using HyperRESEARCH™ and is one example of the seven made. This mind map was the most detailed of the seven and was specifically used to exemplify how findings were organized for thematic analysis. The mind map in this figure was recreated from the original version to allow for a higher-resolution format, but the text within each box was not altered, and none of the other six mind maps were merged into this one.

The research team read through the transcripts to familiarize themselves with the data. A directed content analysis approach involving a hybrid of deductive and inductive coding was used when reviewing the transcripts [13]. The initial coding scheme was pre-tested on the data to mitigate difficulties and variations and informed by the MOBCR framework [5,12]. We engaged in open coding of the data along with analytic memo-taking in a shared document [15]. Using the directed content analysis approach, codes were subsequently grouped and categorized to explore associations and themes between the categories and our framework [12]. Analysis was then performed iteratively until the team agreed that the analysis had adequate conceptual depth; was conceptually plausible, resonated with existing literature; and was externally relevant to the broader academic community [16]. EK then used translational coding to generate seven mind maps of the analyzed data [17]. Central categories within these mind maps focused on topics frequently expressed during participant interviews, such as the advantages of the VL environment (Figure 1). Mind maps were used in our methodology to enhance openness, encourage introspection, and foster discussion to represent connections between various codes visually. Through the use of HyperRESEARCH™ software, the most common codes were identified, which later became the center of a respective mind map with subcategories connected to those primary categories [13,14]. These mind maps subsequently informed thematic analysis.

## Results

**Figure 2:**
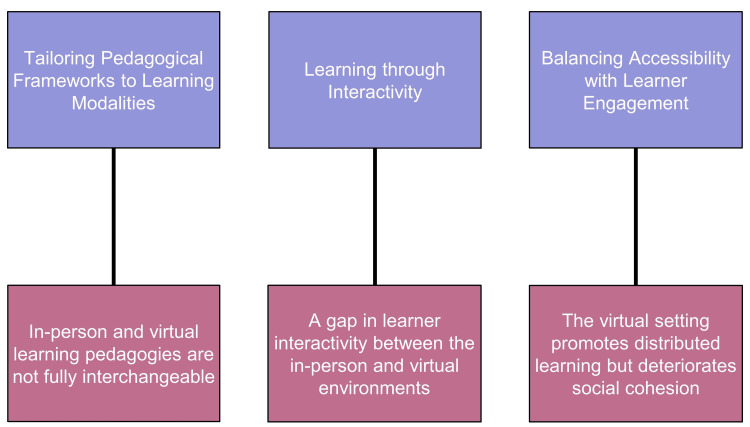
Three themes about clinical reasoning development were constructed from the qualitative analysis, emphasizing (1) tailoring pedagogical frameworks that are often not fully interchangeable between in-person and virtual learning settings, (2) understanding the disparity in interactive learning among different modalities, and (3) balancing accessibility with the potential for decreased learner engagement. The purple boxes represent the three central themes, while the pink boxes provide a more detailed description related to each theme; this diagram was created by the authors of this study.

Interview transcripts from eight first-year medical student participants from McMaster University were analyzed using thematic analysis, and three themes (Figure 2) were constructed: (1) tailoring pedagogical frameworks to learning modalities, (2) learning through interactivity, and (3) balancing accessibility with learner engagement. Extracts of participants’ verbatim statements were used as supporting evidence for the findings that corresponded to each theme.

Tailoring pedagogical frameworks to learning modalities 

Participants strongly emphasized the importance of the resident teacher’s ability to adapt in real time and customize their pedagogical frameworks to the IPL and VL settings to teach CR skills effectively.

Within the IPL setting, participants highlighted the increased utility of relying on non-verbal body language to enable the resident teacher to understand a learner’s comprehension of key CR domains. This was in contrast to the VL setting, where people may not visually see each other if video-calling is not facilitated, requiring a stronger reliance on verbal communication to convey understanding and feedback regarding medical case scenario comprehension. Relying on non-verbal cues within the IPL setting permitted deeper discussions between teachers and participants, promoting CR skill development, particularly in the domains relating to reflecting on case-based medical scenarios, insight into weaknesses, and acknowledging differences in opinions. Using chalkboards and whiteboards in the IPL setting allowed both the teacher and participants to use non-verbal cues while interchangeably writing on them to gauge understanding, whereas this pedagogy was not efficacious virtually:

“In-person, there is a chalkboard or a whiteboard in case the presenter has to demonstrate something.” This participant also commented, “The presenter is able to pick up on your body language, and if you look confused, they might elaborate a bit more. When it comes to virtually, there is really no feedback.” (Participant Four)

Identifying pertinent facts was also more easily facilitated during IPL sessions through the use of physical chalk or whiteboards to present relevant case information. These physical boards provided sufficient space to write detailed notes and allowed participants to see all aspects of a case simultaneously. This comprehensive view promoted a superior understanding of the relationships between different pieces of information to enable more effective CR. In contrast, the virtual setting provided a limited writing space, often requiring scrolling or switching between screens. This fragmented view made it more challenging for participants to maintain a holistic understanding of the case and follow the flow of information seamlessly. Participants cited virtual frameworks that used pre-made reference material as a method to mitigate this issue. These materials could be accessed and reviewed concurrently with the session, allowing for better retention and understanding of the discussed clinical information:

“When we were in-person, we had all the facts of the case on the board, and we could see everything at once. So, the history of presenting illness, symptoms, physical exam, and labs were all in one place. But when we did the session virtually, the whiteboard had to keep moving because it was one screen, and the resident had to draw things, so we could not see all the facts at one time. From that perspective, it was easier in-person because we could see everything and then realize why you may have thought this was heart failure exacerbation, but it was not. These were the mistakes I had made cognitively, but on Zoom, it was just not the same.” (Participant Five)

Additionally, participants frequently cited the use of specific online technologies as methods of tailoring pedagogical frameworks to the virtual setting. Examples included using online search engines and pre-made sets of slides. Teachers providing computer-searchable presentation slides during online sessions allowed participants to reference discussed topics to resources that could be utilized in the future. This enabled CR skills, such as identifying and citing appropriate sources and recording and collecting information:

“In terms of virtual, I really liked that we could have a pre-made set of slides or reference material. I found it especially helpful when we were interpreting something such as an X-ray or if they had specific lab values we were supposed to look at. I found that was really helpful. We were able to develop an approach and work through cases. I think we would not have been able to do that in-person unless there was a lot of assisted technology.” (Participant Three)

Virtual sessions were also more favorable when resident teachers could create a clearer and more structured approach to ensure participants followed the material effectively. Participants appreciated this more within the VL environment, where many participants endorsed a decreased level of attention span compared to in-person sessions. Participants cited examples such as creating online educational quizzes or games to ensure the sessions remained organized when interactivity was diminished virtually. Utilizing these teaching methodologies allowed for stronger CR development through improved problem-solving capabilities in group settings:

“I think group problem solving could theoretically be effective in the virtual setting, but it would require a lot of organization from the resident standpoint; they would probably have to create some sort of Jeopardy game with definitive rules. It would take a lot more effort to make everything very organized and split the class into teams.” (Participant Five)

Learning through interactivity

IPL was favorable over VL among participants in facilitating stronger social interactions, spontaneous communication, and non-verbal comprehension, which improved CR domains, including generating and rationalizing hypotheses and differential diagnoses, group problem-solving, and self-reflection. Participants felt that in-person small group discussions enhanced their ability to express and understand diverse perspectives they may not have previously considered. These factors represented a repeated theme of interactivity being a key component of CR skill development, which was more pronounced in the IPL setting:

“In-person, you have a lot of people contributing, coming up with their own ideas. Sometimes, someone has an idea that you never thought of or no one really thought of. People are just more engaged in-person and willing to speak up.” (Participant Four)

The physical presence of peers and teachers created a more dynamic and engaging environment that promoted interactivity. Participants highlighted how the in-person immediate feedback and real-time discussions fostered a deeper understanding of clinical concepts:

“When everyone is there in-person, it's a lot more personal; you feel more connected. You see that everyone is there to learn. It is harder to get distracted. Sometimes, even the lecturer can call out on certain individuals, which keeps you more engaged.” (Participant Four) 

This was partly due to the ability to read non-verbal cues and body language, which are crucial for effective communication and collaborative learning:

“I think the added benefit of in-person is we often built the case together. Then our preceptor would adjust the case as we either made errors or made the correct decision.” (Participant Three)

Furthermore, IPL allowed for hands-on learning in simulated environments, easier comprehension of learning objectives through superior engagement, and greater access to diverse opinions:

"Virtually, it is a bit hard for every single person to participate in an equal amount and to heed enough information about them to get feedback." (Participant Six)

Several participants brought up the concept of social awkwardness in the virtual setting. They felt it was challenging for teachers to pick up on their non-verbal cues and recognize when they were confused. Providing and receiving immediate feedback and demonstrating awareness of weaknesses through social cues and body language were recognized as key domains to improve upon in developing CR skills:

“When you' are online in a group setting, it is a lot more awkward. It is a lot less comfortable to speak out and identify your weaknesses. Whereas if you are in-person, it is easier to approach someone teaching you one-on-one and feedback or ask for help.” (Participant Two)

The deficiencies in these domains were influenced by the lack of strong group dynamics during VL sessions. Participants felt that strong group dynamics were paramount to hypothesis generation, self-reflection, formulating differentials, and problem-solving, which are all key components of CR:

“I think in terms of the group discussion, it is a bit easier to flow, and there are no mechanisms of muting and unmuting; that challenged the group dynamics a bit. This made it a little bit stiffer to try and work through clinical reasoning as a group.” (Participant Six)

Balancing accessibility with learner engagement

The VL environment offered several logistical advantages, but one particularly highlighted by participants was increased flexibility. Participants could easily fit sessions into their busy academic schedules without needing to commute, allowing easy access to MW as a supplementary learning opportunity. Additionally, the accessibility of technology at home, such as multiple monitors and devices, facilitated more efficient note-taking and immediate access to online resources to strengthen aspects of CR development. Participants seamlessly integrated research and supplementary materials into their study process to enrich their understanding of clinical concepts. This was felt to be superior in VL compared to IPL as the process of citing research and other resources would be more disjointed in the latter due to the decreased use of online resources in real time:

“It was definitely easier with my schedule because with virtual sessions, you do not have to travel there; that was a good part of it. I also have access to a monitor at home and more technology to take notes. It was easier to do that and focus on jotting down notes for the sessions that we had.” (Participant Two)

While contributing to the convenience of VL, these logistical benefits also come with challenges that can initially impede participation. In virtual settings, initial participation can be hindered by a lack of familiarity among group members and the inherent barriers of online communication. The absence of physical presence often makes participants hesitant to unmute themselves or turn on their cameras, leading to a slower start in collaborative activities. This initial discomfort can impede the flow of discussion and delay the establishment of a productive learning environment. However, as participants became more accustomed to the virtual format and built rapport over time, their participation levels tended to improve:

“At first it was more difficult to participate virtually. I think part of that was because people did not really know each other and were uncomfortable unmuting, turning on their cameras, and saying something. Whereas in-person, they are already all there. At the start, it was a bit slower, but then towards the end I feel we were all able to participate still.” (Participant Three)

Furthermore, this initial discomfort among group members interacting with one another negatively affected CR development in the VL setting. Participants suggested a more structured approach to VL that emphasizes key components of CR by including planned moments for active discussion and collaborative problem-solving:

"The virtual sessions are still good and helpful, so they can be continued, but ensuring people have cameras on or having built-in moments for discussion questions for everyone as a group to talk about might help my worsened ability to clinically reason." (Participant Eight)

In contrast, many participants noted that the IPL environment was felt as a whole to be more engaging than the VL setting:

"It was more engaging in person, given that you are actually able to see them. There is a more tangible feel to the learning environment when you are in person and more engaged with others compared to virtually." (Participant One)

Participants suggested that pre-existing group dynamics were crucial in facilitating interactivity during VL sessions. Groups that had been in person together before VL sessions were felt to be more cohesive, decreasing the social awkwardness that often hindered communicating over video calls and thereby benefiting the acquisition of CR skills:

“It was nice because we already met up a couple of times and already had a group dynamic. We knew each other, but if it were all virtual, maybe it would have been less comfortable to acquire those skills together.” (Participant Seven)

## Discussion

This study supports the effectiveness of MW as a blended internal medicine-focused educational platform designed to enhance CR skills among pre-clinical medical student participants through VL and IPL modalities. Thematic analysis of participant transcript data revealed key insights into how different learning environments impact CR development.

The participants’ emphasis on the differences between pedagogical frameworks in the IPL and VL settings highlights that these are not fully interchangeable when aiming to teach CR skills and are modality-specific. VL pedagogies likely necessitate unique efforts and setups, such as breakouts and ice-breakers, to focus on learning outcomes better facilitated by technology platforms, such as shared review of laboratory results and imaging, sharing educational resources, and collecting or recording information [18]. To increase interaction and engagement in both VL and IPL medical education environments, starting each session with an ice-breaker is recommended to break down barriers and foster a sense of collaboration. Ice-breakers are particularly emphasized in VL settings, where interactivity can be more significantly diminished than IPL [18,19]. The session can be broken down into smaller components interspersed with learning activities that use breakout rooms to help learners socialize. Many virtual platforms also have whiteboards, which can be used as a canvas of collaboration for students to contribute to an exercise if accessible to all group members [18].

Constructive alignment, which involves using an outcome-based approach to deliberately match content areas to the ideal modality, could add value to the learning experience. Within this framework, learning objectives, learning activities, and performance assessments should be tailored to one another to optimize the unique advantages that the IPL and VL modalities have to offer CR development [20]. One study illustrated this by creating a virtual emergency department simulation game called EMERGE [21]. Their objective was to expose students to the challenges of prioritizing tasks and assessing the urgency of emergency situations. They found that, compared to small-group problem-based learning, EMERGE was superior in creating simulated pressure and effectively developing CR skills in a controlled, interactive VL environment [21].

The results also underscore the pivotal role of emphasizing interactivity in medical education, with IPL demonstrating a pronounced advantage over VL in this regard. Bandura’s Social Learning Theory posits that learning occurs through observation and modeling within a social context [22]. This involves observing behaviors and the internal cognitive processes that enable learners to understand and replicate those behaviors [22]. Students can gain valuable insights by observing their peers' reasoning and decision-making processes and learning from their own and peers' mistakes and experiences [23]. Prior qualitative studies exploring students’ experiences with online classrooms demonstrate reduced knowledge-sharing behaviors and social learning overall in the virtual setting [24]. There was also decreased feedback from the instructors and their peers, which was echoed by several participants in MW. A scoping review on virtual case-based learning highlighted that students in virtual settings faced challenges with communication and social interaction, which are critical for effective collaborative learning, engagement, and, thereby, CR development. The lack of immediate feedback and difficulty in facilitating group discussions online were significant factors that affected students’ perceived learning and their implicit experience [25].

However, the VL environment provided significant logistical advantages, notably increased flexibility and convenience, which appear to impact components outside participants’ perception of learning objective achievement. These include the implicit benefits of VL in facilitating distributed learning, concomitant clinical obligations, and accessibility. A previous study reported that VL sessions saved students' time and improved their academic performance by optimizing the use of their time [26]. Simply providing recorded lectures as a method of asynchronous online learning allows students to absorb and process course material at their own pace. This flexibility supports individualized learning paces and enhances overall comprehension and retention [27].

Technologies available in the participants’ homes also facilitated enhanced information collection and resource citation, which promoted superior development of these CR domains in the VL setting. Access to digital educational materials encourages students to explore diverse resources and supplement their learning using cited research [28]. This example of self-directed learning draws upon several CR domains described in the MOBCR framework, including collecting information about a clinical case, asking relevant questions, and citing appropriate research sources [5].

Initial participation in VL was hindered by unfamiliarity among group members and online communication barriers. This initial discomfort impeded discussion flow and delayed productive learning. Over time, as participants acclimated to the VL format and developed rapport, participation levels improved, partly alleviating these challenges. This dilemma highlights the importance of facilitating a community of practice, a theory describing a group of people who share a common interest, passion, or concern and interact regularly to improve their knowledge and skills in that area [29]. A community of practice is a model that also allows CR development to flourish and is often more easily established in IPL settings. This suggests a potential model where the IPL setting serves as a prerequisite for developing a community of practice before transitioning to VL sessions, a proposed approach discussed in further detail later [30,31].

Diminished participation in the VL environment can be attributed to a diminished ability to facilitate near-peer teaching. Poor interactivity between ‘peer learners’ and ‘peer teachers’ has been attributed to learners preferring to remain anonymous in the virtual platform and believing that the learning process should be a unidirectional transmission of knowledge from teacher to learner [7,32]. Near-peer teaching can also help facilitate socialization to healthcare contexts, improving CR [30]. Collaborative learning is superior to individual learning in fostering CR by filling in knowledge gaps and counteracting flaws in reasoning [30]. CR is not solely a cognitive process but also a contextually situated and socially mediated activity [30]. Learning in a community of practice, where students can observe and interact with peers and supervisors, helps them handle complex clinical situations, ask critical questions, and test their thoughts against others, ultimately improving their CR skills [30].

**Figure 3:**
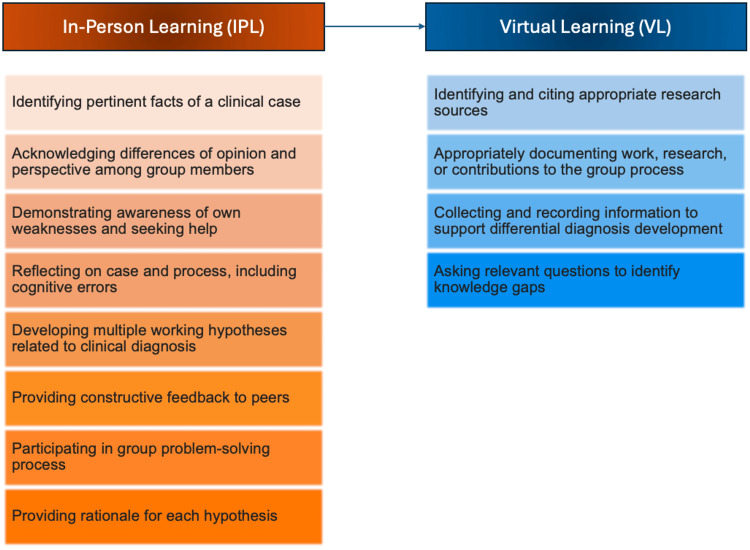
This study’s proposed scaffolding approach for future blended medical education platforms focused on clinical reasoning, which starts with in-person learning and is followed by virtual learning. Evidence from this study is used to determine which corresponding clinical reasoning domains are best developed in each respective modality to emphasize the tailoring of pedagogical frameworks. The clinical reasoning skills highlighted in orange correspond to those best developed in the in-person learning environment, while the skills in blue are best fostered in the virtual learning environment. This diagram, created by the authors of this study, utilizes the clinical reasoning domains from the Milestones of Observable Behaviors Clinical Reasoning framework [5].

Our findings suggest implementing a scaffolding approach to align with a learner’s zone of proximal development through initial IPL sessions that better facilitate social cohesion, followed by VL sessions [10,11]. The scaffolding approach was initially introduced to describe how educator support is gradually adjusted as students become more independent in their learning process [10]. This approach can be used to align with a learner’s zone of proximal development and focus on learning objectives and pedagogies that are tailored to specific domains of CR (Figure 3). This socio-cultural theory is defined as the gap between what a learner can achieve independently and what they can accomplish with guidance from more capable peers [10,11]. Teachers play a crucial role in assisting with tasks beyond the student's current abilities, gradually reducing support as the student develops competency [11].

A blended model that begins with IPL, followed by VL, may facilitate and allow for the competency of CR domains that require a community of practice development, near-peer teaching, and socialization to healthcare contexts within the IPL environment to be internalized and carried forward to the VL setting [7,29,30]. These benefits may then overcome the barriers to CR development described in VL settings alone when the support of a physical presence and structured support is gradually reduced. This model could allow for the unique benefits of CR development in both the IPL and VL environments while reducing the disadvantages of VL.

Strengths and limitations

To our knowledge, this is the first qualitative study to investigate and compare the development of CR skills in pre-clerkship medical students within small group virtual and in-person settings. The results from this study will provide insight into the feasibility of teaching CR skills in virtual small-group sessions for medical education researchers and those developing medical curricula. These results will also help educators understand how learners perceive virtualization in the context of CR development and help determine the feasibility of a hybrid IPL and VL model for future learning opportunities in undergraduate medical education within a post-coronavirus 2019 pandemic era. However, it must be acknowledged that this is a small study conducted at a single institution, focused specifically on internal medicine, and involving medical students only at a first-year level, limiting our findings' generalizability. Students in this study were strategically chosen at the end of their pre-clerkship when they are beginning to bridge the gap between theoretical knowledge and practical application and are, therefore, most receptive to developing CR skills. The impact of MW may manifest differently in higher-year medical students.

Future research should investigate blended CR models across different stages of medical education, including clerkship and postgraduate training, to assess their applicability and effectiveness in diverse educational contexts. Additionally, incorporating perspectives from medical educators could provide a more comprehensive understanding of the strengths and limitations of similar platforms.

## Conclusions

This study’s MW-blended platform offers insight into strategies to enhance specific domains of CR among pre-clerkship medical students both in VL and IPL settings. Future blended CR medical education initiatives should focus on modality-tailored pedagogies, emphasizing group interactability and weighing the accessibility of VL against decreased engagement. Educators should ensure that the VL environment preserves social cohesion from IPL settings to prevent barriers to participation and maintain group interaction as a central objective of educational sessions to facilitate CR skill development. Similar future initiatives might benefit from a scaffolding approach, starting with IPL sessions to establish social cohesion and then transitioning to VL sessions to better align with the learner’s zone of proximal development. Scaffolding approaches may optimize CR domains that are best acquired through VL or IPL to emphasize tailoring of pedagogical frameworks. Future research should focus on the long-term impacts of such blended learning models on CR proficiency and explore strategies to enhance VL environments' effectiveness in medical education.

## Appendices

Appendix A: interview questions

**Table 1:** Interview questions for the study participants

Part 1: General Information, Mock Wards Attendance, and Clinical Experiences
1. What year of medical school training are you in currently and what year were you in during Mock Wards?
2. How many in-person and how many virtual sessions of Mock Wards were you able to attend?
3. How many weeks of clinical rotations have you completed since finishing Mock Wards? Which rotations were these?
Part 2: Extent to Which the Virtual Learning Session Reflected Processes by Which Clinical Reasoning Are Obtained During In-Person Teaching Sessions
1. Clinical reasoning has been defined as a “skill, process, or outcome wherein clinicians observe, collect, and interpret data to diagnose and treat patients.” This definition can be repeated if you need it at any time. We want to ask you about your experience learning clinical reasoning, especially comparing the in-person versus virtual sessions. Were you in pre-clerkship or clerkship at the start of Mock Wards? How would you describe your experience with developing clinical reasoning skills throughout your medical education thus far?
2. Did you find Mock Wards beneficial as a supplementary learning resource towards developing your clinical reasoning skills?
3. How do you feel about the virtual sessions compared to the in-person sessions regarding the acquisition of clinical reasoning skills? Which aspects were similar? Which aspects were different? Which aspects were advantageous? Which aspects were disadvantageous?
Part 3: How Well Virtual Learning Allowed for Group Problem-Solving
1. Did the virtual learning environment benefit, impair, or make no difference in your ability to participate in small group sessions?
2. Do you feel group problem-solving could take place effectively in the virtual setting? Do you feel problem-solving was superior, equivocal, or inferior to the in-person setting?
3. Did group problem-solving play a large or small role in your development of clinical reasoning in the in-person vs. the virtual setting?
Part 4: Challenges and Barriers to Learning Within the Virtual Context
1. How do you feel virtual learning can best benefit your ability to develop clinical reasoning skills?
2. What challenges did you find in the virtual setting that were not present in-person pertaining to the acquisition of clinical reasoning skills?
Part 5: How the Process of Developing One or More Domains of Clinical Reasoning Was Impacted by the Virtual Context
1. For the subsequent domains of clinical reasoning I mention for the remainder of this last section of the interview, please describe if the virtual context was beneficial, similar, or inferior compared to the in-person sessions and why. The first clinical reasoning domain is the identification of pertinent facts in a clinical case scenario.
2. Collection of data regarding creating a list of differential diagnoses.
3. Creating a hypothesis related to a potential diagnosis.
4. Ability to provide an explanation for hypothesis created.
5. Ability to give peers constructive feedback.
6. Asking relevant questions to fill gaps in your knowledge relating to solving the case problem.
7. Ability to cite research relating to the cases.
8. Reflect on the clinical case and the process to determine cognitive errors you may have made.
9. Ability to seek insight into your own weaknesses and ask for help regarding these.
10. Ability to identify differences in opinion and thought processes among group members.
11. Ability to document your own and others’ work, ideas, or notes.
